# Reviewing the limb apraxia concept: From definition to cognitive neuropsychological models

**DOI:** 10.1590/S1980-57642010DN40300004

**Published:** 2010

**Authors:** Joana Mantovani-Nagaoka, Karin Zazo Ortiz

**Affiliations:** 1 Speech Therapist, Masters in Science, Department of Speech Pathology, Universidade Federal de São Paulo (UNIFESP) São Paulo SP, Brazil; Speech Therapist in APS Santa Marcelina, OS Itaim Paulista, São Paulo SP, Brazil.; 2 Speech Therapist, PhD in Neuroscience from UNIFESP, Professor at the Department of Speech Pathology and Audiology; Department of Speech Pathology and Audiology, UNIFESP, São Paulo SP, Brazil.

**Keywords:** apraxias, neuropsychology, apraxia, ideomotor

## Abstract

Apraxia is a disorder of learned skilled movements, in the absence of elementary motor or sensory deficits and general cognitive impairment such as inattention to commands, object-recognition deficits or poor oral comprehension. The first studies on apraxia were performed between the late 19th and early 20th centuries, however controversy remains in praxis literature concerning apraxia types, neuroanatomical and functional correlates, as well as assessment and treatment of apraxia. Thus, a critical review of the literature was conducted searching the literature for evidence contributing to a more detailed description of apraxia and its clinical patterns, physiopathology and clinico-anatomical correlations, as well as apraxia assessment.

## Abstract (pt)

A apraxia é um distúrbio da realização de gestos ou atos motores aprendidos sem que haja anormalidades em canais sensoriais aferentes ou motores eferentes, deterioração intelectual, nem alteração de atenção ou de compreensão dos comandos verbais. Os estudos sobre a apraxia iniciaram-se entre o fim do século XIX e início do século XX, no entanto, a literatura ainda apresenta controvérsias no que se refere à classificação desse quadro, seus substratos neuroanatômicos e funcionais, bem como suas formas de avaliação e tratamento. Assim, o presente estudo realizou uma revisão crítica da literatura visando buscar evidências científicas que contribuam para melhor delinear a apraxia e suas formas de manifestação clínica, sua fisiopatologia e as correlações anátomofisiológicas, bem como suas formas de avaliação.

Apraxia is defined as a disorder of learned skilled movements, in the absence of elementary motor or sensory deficits and general cognitive impairment such as inattention to commands, object-recognition deficits or poor oral comprehension.^1,2^

Liepmann was one of the first scholars to describe apraxia in its currently recognized form when, in 1905, he defined it as a unitary phenomena characterized by a range of clinical manifestations stemming from different levels of dysfunction in the same process of motor action production.^3^

However, controversy remains in praxis literature concerning apraxia types, neuroanatomical and functional correlates, as well as assessment and treatment of apraxia.^4^ Thus, the aim of the present study was to search the literature for evidence contributing to a more detailed description of apraxia and its clinical patterns, physiopathology and clinico-anatomical correlations, as well as apraxia assessment.

To this end, a critical review of the literature was conducted which entailed a search for scientific articles indexed on the following databases: Lilacs, Medline, Pubmed and ScienceDirect. The research strategy adopted involved a search using the keywords of *apraxia*, and combination of *apraxia* with *aphasia* and *language*, in the fields of descriptors, words contained in titles and/or summaries, for articles published up until May 2009.

## Definition and physiopathology of apraxia: conceptual or motor deficit?

The first studies on apraxia were performed between the late 19^th^ and early 20^th^ centuries. One of the pioneers in the studies on apraxia, Liepmann, was the individual who in 1905, first used the term ideomotor apraxia to describe the inability to correctly transform the desire for intended movement into appropriate motor action, associating apraxia to a disorder in the execution of movements, and not to a failure in gesture (symbolic) evocation.^3^ He described 83 clinical cases of brain damage, classified as either left- or right- hemisphere lesioned subjects, based on the side of hemiplegia. Liepmann noted that 20 of the 41 right-paralytic cases, namely, those with probable left-hemisphere damage, presented disturbances of praxis performance, whereas the group of 42 left-paralytic cases did not present the same difficulties. Although all patients were also aphasic, they still showed impairment in tasks of execution under imitation, ruling out the notion that failures in executing movements occurred due to comprehension deficits. These results led Liepmann to postulate that the brain’s left hemisphere was responsible for skilled movement planning of both right and left arms, while corpus callosum mediation occurred in this latter limb. Geschwind, in 1965, also attributed control of motor actions to the left hemisphere. He suggested that to correctly pantomime in response to verbal commands, the verbal information must be processed by the auditory pathways and posterior language areas located in the left temporal lobe, subsequently flowing to the ipsilateral motor association cortex in right-handed subjects. By contrast, in left-handed individuals the information must flow to the contralateral motor association cortex via the corpus callosum. Therefore, lesions to the supramarginal gyrus or arcuate fasciculus would result in apraxia characterized by comprehension of verbal commands, but with an inability to execute them due to disconnection of the posterior language areas from the anterior motor association area. Gesture imitation would also be affected due to the disruption between visual and motor areas (arcuate fasciculus). The actual use of objects however, should remain intact.^5^

However, De Renzi, Pieczuro and Vignolo, three years later and contrary to postulations by Geschwind,^5^ described deficits in these abilities in their left-hemisphere damaged patients, particularly among the aphasics, when investigating the ability of healthy and brain damaged individuals to actually use objects. Moreover, they found a strong correlation between praxic and auditory verbal comprehension scores leading them to conclude that, besides being specifically associated with lesions of the dominant left hemisphere, particularly underlying language areas, apraxia appears to be related to a specific conceptual disorder, typically found in aphasia.^6^

**Figure 1:**
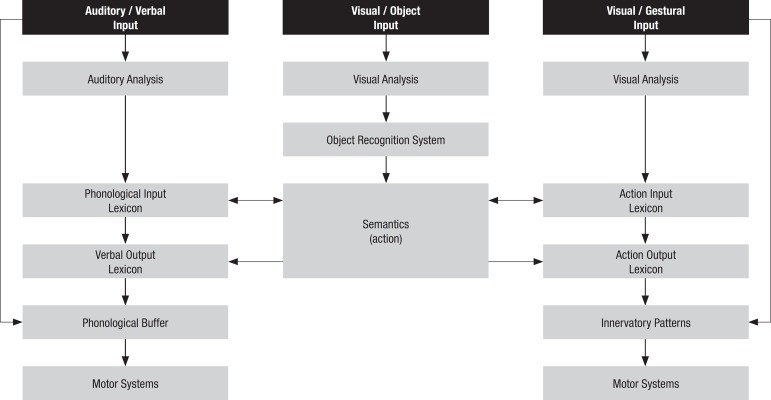
Model of Limb Praxis proposed by Rothi, Ochipa and Heilman.^3^

Following this insight, an increasing number of studies have sought to investigate the existence of conceptual deficits in apraxic disorders. Drawing on numerous case reports and reports by Liepmann on apraxia, Rothi, Ochipa and Heilman proposed a model of limb praxis (Figure 1), in which the process of motor action production involves a conceptual component in the production of intended movements. In other words, the process of gestural production includes both conceptual and motor production systems, with interaction of lexical-semantic and action processing during the execution of praxic movements. Any disruption in this process could lead to deficits in the execution of learned movements, causing the spectrum of clinical manifestations of apraxic disorders.^3^

In the proposed model, the conceptual system comprises lexical and semantic components, as in the models of language processing, related to three types of knowledge:

1. Instrument and object function, where instruments are objects used to provide mechanical advantage to action and objects are the elements which receive this action;
2. Knowledge on independent object actions; and
3. Knowledge on the organization of simple actions into sequences.

The motor production system on the other hand, is composed of the sensory-motor component of action, which includes the information on programs as well as their translations into actual action.

The model inherited the term lexicon, originally used in language studies, which refers to the part of the language system that provides a processing advantage for words that the language user has had prior experience and uses it as the internal representation of learned movements, thus corresponding to the “movement memories” proposed by Liepmann.

Hence, the model is composed of input and output action lexicons, owing to the dissociations described between gestural comprehension, production on command and through imitation. The input action lexicon would thus be responsible for recognition of previously learned gestures, while the output action lexicon would be responsible for the production of these gestures.

Since the publication of this model, many subsequent studies have been based on these assumptions, ranging from investigations into the conceptual correlations involved in motor action processing, to attempting to understand the nature of different apraxic clinical manifestations, enabling more accurate diagnosis of limb apraxia types.

In 1995, for example, Goldenberg carried out a study whose aim was to investigate the role of a general concept of the human body position and configuration in apraxic disorders, independently of whether a person’s own body is concerned or not. The author concluded that even meaningless gestures involve semantic conceptions in so far as they represent conceptual knowledge about the human body.^7^

Indeed, many years later, in 2002, Goldenberg and Strauss also found correlations between conceptual knowledge of the body and gestural execution, ascribing this semantic processing to the brain’s left hemisphere.^8^

In a further bid to corroborate that limb apraxia, beyond being a motor execution disorder, involves deficits in the conceptual level of movement stemming from damage to brain left-hemisphere, Goldenberg, Hermsdörfer and Spatt conducted another study, in which they investigated the kinematics of movement trajectories of imitations of meaningless gestures. The aim was to assess the internal preprogramming of skilled movements of either hand, through computerized analysis of movements performed by both healthy control individuals and brain right- and left-hemisphere damaged patients.^9^

The majority of participants with left-hemisphere damage presented non-fluent and hesitating movements, suggesting feedback-controlled movement. A dissociation pattern was also observed in this group, in which some patients showed a totally normal kinematic profile, but also presented incorrect end-positions. The authors concluded that the disorder occurs predominantly in the presence of left-hemisphere brain damage and is related to a failure in determining the target position rather than the execution of the movement. Feedback control appears to be a compensatory strategy rather than the source of apractic errors.

## Apraxia: neuropsychological models and clinical patterns

In the context of attempts to characterize specific apraxic pictures caused by selective deficits in the different components involved in the gestural production process, Ochipa, Rothi and Heilman described the case of a left-handed patient with damage to the right-hemisphere who presented deviations in the actual use of instruments, both during assessment situations as well as in natural settings. The patient’s language abilities were evaluated and praxis testing carried out using the same set of stimuli for all the tasks. These tasks included object identification by name and function, their naming and oral function definition, actual use of instruments and objects, instrument selection and pantomime in response to commands and by imitation. The patient presented a picture that the authors defined as ideational apraxia, characterized by the inability to correctly use actual instruments and objects, yet with better performance by imitation. They believed the deficit to be related to failure in accessing the knowledge about instrument function, since naming and name recognition abilities were spared, although the patient was unable to define or point out an instrument based on its functional definition.^10^

However, the same authors in 1994 described another clinical case of a left-hemisphere damaged patient who had been clinically diagnosed with Conduction Aphasia and whose performance on pantomime in response to verbal command was superior to pantomime imitation, yet with spared comprehension of these gestures. They called this clinical picture Conduction Apraxia, drawing parallels with language and aphasia studies. The input and output action lexicons were also found to be spared evidenced by the patient recognizing pantomimed gestures, and also being able to use actual objects correctly. The difficulties pantomiming in response to verbal command coupled with poor imitation of these gestures however, suggested additional difficulties stemming from disruption in gestural processing between the input and output action lexicons.^11^

Adopting the same terminology, Politis described another case of Conduction Apraxia. A 51-year-old patient, following a traumatic brain injury, presented Wernicke’s Aphasia and ideomotor apraxia with a disorder pattern, characterized by an inability to imitate familiar and non-familiar gestures. The other praxic abilities assessed, including tool use and gestural execution by means of object visual input, were all spared. The author believed the deficits to be related to disruptions in the pathway connecting both input and output action lexicons.^12^

**Figure 2:**
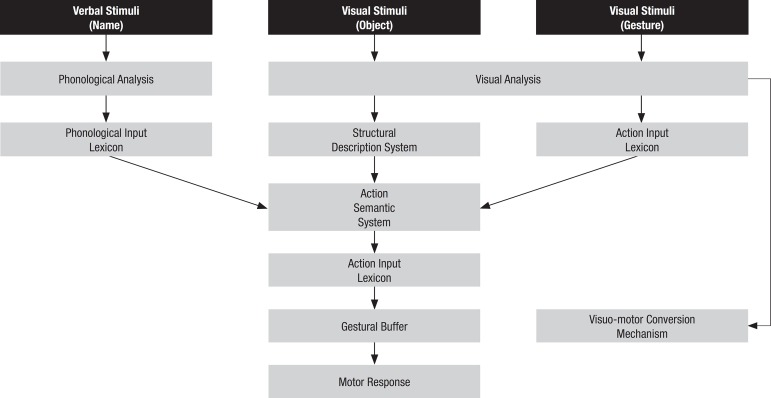
Modified model of limb praxis (Cubelli et al, 2000).

In a bid to investigate and describe the clinical pictures attributable to specific disturbances in each of the components of limb praxis processing, Cubelli et al. revised the original model of limb praxis, suggesting several modifications. The revised version of Rothi et al.’s model of limb praxis incorporates a visuomotor conversion mechanism, devoted to transcoding visual analyses into motor programs. Another difference to the original model is that no direct link between input and output lexicon is assumed. In addition, the lexical and non-lexical routes are thought to converge in a gestural memory buffer, whose purpose is to hold a short-term representation of the motor program to be executed. The “innervatory patterns” proposed by Liepmann and encompassed in Rothi et al.’s model were also dropped from the revised model. The revised model of limb praxis is shown in Figure 2.^13^

Envisaging five different clinical patterns of apraxia, the authors sought to assess the model by searching for these predicted patterns of spared and impaired functions in a group of 19 patients with left hemisphere damage and in a group of 20 healthy participants.

Besides neuropsychological and language assessments, participants were submitted to praxic tasks such as production of transitive and intransitive gestures in response to verbal commands and by imitation. Subjects were also submitted to transitive gesture recognition assessment in which they were instructed to identify, among four pictures presented, the image corresponding to the correct use of a familiar object with the aim of assessing access to the input action lexicon and its connection to the semantic system. Only one of the five predicted patterns of limb apraxia was not observed in this study, although this has been described in other studies,^11,12^ in which the deficit was limited to the visuomotor conversion mechanism. The authors nevertheless, described four other clinical patterns: one case suggesting failure in the input action lexicon, since the difficulty was limited to gestural recognition; a case of a patient that presented deficits only in gestural production on command and in the actual use of objects, suggesting failure in the output action lexicon; three cases of failures of the gestural buffer; one case of deficit in the actual use of objects and in the recognition of transitive gestures, suggesting failures in the action semantic system.

Interestingly, another clinical pattern found in this study suggested dissociation between transitive and intransitive gesture processing. One of the cases studied demonstrated failure in the output action lexicon, limited to the production of transitive gestures, while the production of intransitive gestures was spared.

Several studies have shown evidence that motor action processing may be dissociated according to the type of gestures, in terms of its semantic features (meaningful or meaningless gestures) as well as its relationship with instruments and objects (transitive and intransitive gestures), producing dissociated patterns of apraxic disorders.

Bartolo et al. for example, described three clinical cases of apraxic patients. Two of them presented performances comparable with healthy participants in gestural production on command and in meaningful gesture imitation tasks. However, they did show a selective deficit in the imitation of meaningless gestures. This pattern suggests impairment of the visuo-motor conversion mechanism and corresponds to the clinical picture known as “conduction apraxia”. The third case, however, presented with the opposite pattern, showing impairment in meaningful gesture production both on command and imitation, combined with normal performance in the imitation of meaningless gestures, which suggests a spared conversion mechanism as well as gestural buffer. The impairment therefore seems to be limited to the lexical route, although the input action lexicon and the semantic system appear to be spared, since the patient performed well on tasks of gestural recognition and identification. Accordingly, the conclusion was drawn that the disorder must be related to failures in the output action lexicon or access to it. These contrasting profiles indicate a double dissociation between lexical and non-lexical routes of gestural production, leading to dissociated patterns of deficits related to meaningful and meaningless gestures.^14^

In terms of selective deficits in transitive and intransitive gestures, Hanna-Pladdy et al. investigated error patterns in right- and left-hemisphere damaged subjects and healthy controls in the execution of transitive and intransitive gestures on command. The hypothesis was that differential error patterns within left and right hemisphere damaged patients might reflect the relative contribution of each hemisphere to praxis functions. The left hemisphere damaged group was found to produce more qualitative errors than the right hemisphere damaged group, confirming the hypothesis of left hemisphere dominance for praxic functions. Nevertheless, the error type analysis suggested bi-hemispheric representation of specific spatial and temporal aspects of skilled movements, although the left hemisphere seems to be dominant in the representation of action semantics and spatiotemporal movement representations. The right hemisphere, on the other hand, seems to play a role in timing associated with the spatial properties of movements produced, corroborating previous findings of Goldenberg and Strauss.^8^ Concerning gesture type, only the left hemisphere damaged subjects showed impaired intransitive gesture execution. These subjects did however, experience greater difficulty executing pantomimed gestures involving the use of objects, which coincidentally has been investigated in several recent studies.^15^

However, should pantomimes be considered transitive or intransitive gestures? Bartolo et al. called attention to this ambiguity over pantomimes of object use because they could indeed be regarded as transitive gestures given they involve conceptual features of object use to some degree. Nevertheless, these objects are not physically present, which could lead us to deem them intransitive gestures. The authors also stressed that, although they could be similar in some contexts, pantomimes of object use and symbolic gestures describing object use should not be confused, because in some instances they differ from each other, as is the case of scissors. Given pantomimes are rarely performed in everyday life they could be considered novel gestures which, as such, do not have a previous motor program available in long-term storage.^16^

The correct pantomimed gesture has to be performed considering distance, configuration and orientation of the acting hand with regard to the object features and the subject’s body. Spatial and postural errors are frequently observed in apraxia. Therefore, the authors hypothesized that when performing pantomimes of object use, the semantic features regarding function of the object stored in the action semantic system, and the motor program for its use stored in the output action lexicon, should be integrated and used to plan the motor act, where the whole process is made possible through the working memory.

In the same study, the authors hypothesized that failures in working memory could explain the selective deficit seen in pantomime production, which led them to suggest a review of the model of limb praxis to include a creative mechanism which integrates and synthesizes perceptual inputs, together with information made available from the semantics and output lexicons, to generate new motor programmes.

Hence, this refined version of the cognitive model of limb praxis includes three different routes, each responsible for different gestural categories: the lexical route, for the processing of meaningful transitive or intransitive gestures; the non-lexical route, for the imitation of meaningless gestures; and a third pathway, centered on the workspace that allows performing and imitating pantomimes.

Schnider et al. also reported that brain damaged individuals presented greater difficulty performing pantomimed gestures, although they ascribed this failure to the complexity of the motor act, as opposed to its implicit conceptual features.^17^

Concerning the error types evident in pantomime tasks, perhaps the most frequently described is the so-called “body part as a tool” (BPT), which highlights the importance of giving specific instructions when assessing pantomime performance.

Raymer et al. thus investigated the performance of a group of left hemisphere damaged subjects and two groups of healthy controls on a pantomime production task. All participants were submitted to the same task, whereas only one of the healthy control groups and the left hemisphere damaged group were reinstructed to modify the inappropriate BPT responses when they occurred, while the other group of healthy controls were not reinstructed. Although this type of error occurred in both brain damaged and healthy control subjects, only the controls that were reinstructed were able to successfully modify their BPT errors, where left hemisphere damaged subjects proved unable to modify their responses. This finding suggests that even in cases involving culturally accepted, emblematic gesture which are more easily evoked when performing pantomimes, only healthy subjects are able to modify their responses and correctly perform the pantomime, while left hemisphere damaged subjects continue to present the pathological error pattern.^18^

However, according to Duffy and Duffy (1989), the BPT error type cannot be considered as pathognomonic for brain damage, since in their study they found no significant difference concerning this type of error between left-or right-hemisphere damaged subjects and healthy controls.^19^

## Anatomophysiological correlations

Hermsdörfer et al. investigated the neural correlates of pantomimes and actual tool use in healthy subjects using an event-related functional magnetic resonance imaging (fMRI) paradigm. The subjects were requested to demonstrate the use of various tools, either as pantomimes or with the actual tool held in each hand. Both pre-movement and movement events were evaluated. The same neural substrate was activated during visual analysis, followed by movement planning and preparation, during both pantomime performance and actual tool use. Bilateral superior parietal lobe, right intraparietal sulcus and right temporal lobe activations were observed during preparatory phases of both conditions. However, more intense activation of different cortical structures was found during actual tool use than on performing of pantomimes. Therefore, the primary sensorimotor areas, as well as cerebellum, basal ganglia and thalamus, were more intensely activated when the subject had the tool in their hands, probably due to the more intense sensory stimulation through skin contact with the tool and owing to the stronger and more precise control of finger and hand movements needed. Nevertheless, there was also more intense activation of cortical areas involved in higher aspects of motor control in the temporal, posterior parietal and inferior frontal lobe, where this more intense activation was more prominent in the right hemisphere. These findings suggest left hemisphere dominance for the pantomime condition yet more symmetrical activation during actual tool use.^20^

Akin to the study by Hermsdörfer et al., other scholars have sought to investigate the neural substrate along with the most susceptible brain areas which, when damaged, lead to deficits in limb praxis processing.

Moll et al. investigated the performance of healthy subjects while performing pantomimes of object using each hand on functional magnetic resonance imaging (fMRI). They found that, irrespective of the hand used, left intraparietal sulcus activation took place in all subjects, suggesting that lesions in this specific area could be linked to conceptual apraxia.^21^

Similar results were found by Buxbaum et al. who described deficits in transitive gestural production among patients with left inferior parietal lobe damage, whereas patients with bilaterally frontoparietal damage showed difficulty performing meaningless gestures.^22^

The authors believed these findings could be related to the role played by each of these cortical structures during gestural processing, attributing the mediation of representations of familiar skilled hand-object interactions to the left inferior parietal lobe.

On the other hand, dynamic adjustments related to hand posture and movement during any kind of action might be mediated by frontotemporal structures bi laterally, where damage to this structure could affect meaningless gestures.

Aiming to draw parallels between brain areas involved in praxic movements and the different types of apraxia described, Wheaton and Hallet carried out an extensive review of the literature on the theme and reported that parietal damage was more strongly related either to conceptual apraxia, characterized by loss of instrument or tool concepts leading to inappropriate object use, or to ideational apraxia in which failures in the sequence of movement steps could present order inversion or missing steps.^4^ It is important to emphasize that controversy remains in the literature over apraxia type classification, since these authors distinguish conceptual and ideational apraxias, while Ochipa, Rothi and Heilman called these deficits in accessing knowledge on tool function, ‘ideational apraxia’.^10^

Furthermore, according to Wheaton and Hallet, after accessing the instrument’s function concept and its manner of use, the implementation of this formula into motor action takes place, where temporal and spatial organization of action is mediated by premotor areas. Thus, damage to areas interconnecting parietal and premotor lobes would be associated with an inability to perform pantomimes, imitate them or even use objects appropriately, possibly leading to errors concerning spatial orientation, slow movements, movement amplitude as well as failure in performing communicative gestures, consistent with ideomotor apraxia. The BPT error type may occur in these cases.^4^

Finally, motor commands should be correctly executed by cortical motor areas, where lesions would lead to loss in refined and precise movements of the hands and fingers along with impaired basic motor coordination, in a picture known as limb-kinetic apraxia. The authors believed that in order for successful performance of transitive gesture to occur, intercommunication is needed between all the structures involved in all steps of movement: planning, programming and execution of motor action.

Hence, there seems to be a common denominator in the literature concerning the relationship between the parietal lobe and semantic processing of gestures, more specifically the left inferior parietal lobe, particularly related to object and tool use. Frey described findings that confirmed this relationship, concluding that the inferior parietal lobe seems to play a critical role in the integration of semantical information, processed by the occipitoparietal ventral stream, into sensorimotor information derived from the occipitoparietal dorsal stream during tool use.^23^

## Apraxia assessment

Regarding methods of assessing limb praxis, besides the cognitive models of limb praxis which postulate several components involved in gestural processing and shed light on the function of these components characterizing the clinical repercussions of these deficits, few studies have focused on the clinical methods of assessing limb praxis.

Wheaton and Hallet pointed out the absence of standard batteries for the assessment of this disorder, which in turn lead to a lack of homogeneity among different studies. They concluded that a thorough investigation of limb apraxia should include at least tasks to assess pantomime in response to verbal command, imitation, transitive gesture production in response to visual input of the object, actual object use, and performance in real situations, of both transitive and intransitive gestures. Furthermore, they suggested investigation of pantomime recognition and discrimination, production of meaningless gestures and tasks of tool selection. Lastly, they believed that assessment of basic motor control, which should remain intact, would be extremely valuable in praxia testing.^4^

Another relevant aspect that warrants attention when evaluating praxic abilities is the influence of demographic features, which might interfere in individual performance. Nevertheless, few studies to date have specifically investigated the influence of such variables on limb praxis.

Chipman and Hampson, for example, investigated the presence of sex-related differences on a multiple meaningless gesture sequence tasks involving both hand and arm movements in healthy subjects. They described a female advantage for movement accuracy and speed of execution.^24^

With regard to the demographic variable of age, Pedersen et al. found manual apraxia to be associated with increased age in patients following acute stroke. However, they only assessed the production of three intransitive meaningful gestures to command (pointing, waving and greeting), whereas other praxic abilities were not investigated.^25^

Regarding the influence of schooling on praxic abilities, few studies have directly investigated the influence of years of schooling on individual performance in praxia batteries. This can be explained by the fact that almost all studies on apraxia have been conducted in countries whose schooling levels are largely homogenous.

Okamoto however, investigated the performance of Brazilian healthy elders on a battery for praxis assessment composed of tasks to evaluate ideomotor and ideational praxis, symbolic gestures, meaningless gestures imitation and constructive praxia. He found age to influence only the imitation of meaningless sequencing gestures, while schooling had greater impact and influenced other tasks.^26^ Nitrini et al. also described schooling effects on Luria’s fist-edge-palm test in a Brazilian population.^27^ Ardila et al. reported that motor abilities related to the reproduction of meaningless gestures, sequential and alternating, were influenced by schooling, but affected little by differences in age.^28^

## Conclusions

The literature generally considers apraxia to be a disorder of intended motor gestures. There is scientific evidence of semantical processing inherent to limb praxic movement processing, especially concerning tools and object use concepts, which can be attributed to the left hemisphere, more specifically to the parietal lobe.^8,15^ Methods of assessing limb praxis remain diverse, where no consensus exists among the several studies examined. However, there does seem to be a trend toward evaluating those types of gesture predicted in the models of limb praxis, including transitive and intransitive gestures, pantomimes and actual use of objects, in response to verbal command and by imitation. Several apraxia clinical patterns have been described but controversy remains in the literature over apraxia type classification.
